# Polyketides with a 6/6/6/6 Oxaphenalene Pyranone Skeleton from Marine-Derived *Streptomyces* sp. HDN150000

**DOI:** 10.3390/md23050188

**Published:** 2025-04-27

**Authors:** Xiaoting Zhang, Falei Zhang, Wenxue Wang, Xingtao Ren, Tianjiao Zhu, Qian Che, Dehai Li, Guojian Zhang

**Affiliations:** 1 Key Laboratory of Marine Drugs, Chinese Ministry of Education, School of Medicine and Pharmacy, Ocean University of China, Qingdao 266003, China; 2 Laboratory for Marine Drugs and Bioproducts, Qingdao Marine Science and Technology Center, Qingdao 266237, China; 3 Sanya Oceanographic Institute, Ocean University of China, Sanya 572025, China; 4 Marine Biomedical Research Institute of Qingdao, Qingdao 266101, China

**Keywords:** actinobacteria, polyketide, cytotoxic activity

## Abstract

Three new structures named naphpyrone I–K (**1**–**3**) that contain a 6/6/6/6 oxaphenalene pyranone skeleton were isolated and purified from a marine-derived *Streptomyces* sp. HDN155000. Their chemical structures, including configurations, were elucidated by extensive NMR, MS, single-crystal X-ray diffraction, theoretical NMR calculations, DP4+ probability analysis, and ECD analyses. Naphpyrone K (**3**) showed cytotoxic activities against L-02, K562, NCI-H446/EP, MDA-MB-231, and NCI-H446 cancer cells with IC_50_ values of 5.13, 3.34, 2.50, 2.61, and 2.20 μM, respectively. These findings highlight the potential for screening and developing therapeutic drugs from aromatic polyketides derived from marine actinobacteria.

## 1. Introduction

Marine actinobacteria, a branch of actinobacteria, is widely distributed in various ecosystems in the marine environment, including seafloor sediments, rock surfaces, and seawater [1]. Compared to terrestrial actinobacteria, marine actinobacteria are more tolerant of hypoxia, oligonutrition, high salt, high pressure, low temperature, and other unusual living circumstances, which is closely related to their long-term survival in special environments [2]. This group of microorganisms exhibits a complex and elaborate cycle of morphological differentiation. Under suitable environmental and nutrient conditions, the mycelium of marine actinobacteria exhibits multinucleate, branched, slender, and non-septate characteristics [3]. Morphological differentiation in marine actinobacteria is frequently accompanied by complex physiological changes and the generation of a large number of metabolites [4,5]. In recent years, a variety of structurally diverse secondary metabolites with unique bioactivities originating from marine actinomycetes have been exploited [6,7]. Subramani summarized 167 new bioactive compounds produced by marine actinobacteria with antimicrobial, antitumor, anthelmintic, and antimalarial activities, of which the genus *Microcystis* is the richest source of chemical diversity and unique bioactivities [8]. Chen summarized 536 compounds isolated from marine actinomycetes, of which alkaloids (37%), polyketides (33%), and peptides (15%) accounted for the largest proportion, and *Streptomyces* (68%), *Aeromonas* (6%), and *Nocardia* (3%) were the major producers of secondary metabolites [9]. Some researchers have even begun to focus on the effect of marine *Streptomyces* secondary metabolites on drug-resistant bacteria. Cho extracted a new chromomycin from the marine-derived *Streptomyces* sp. MBTI36 with potent antimicrobial activity against Methicillin-resistant *Staphylococcus aureus* (MRSA) [10]. In addition, a study has isolated a novel tirandamycin with vancomycin-resistant *Enterococcus faecalis* inhibitory activity from the marine *Streptomyces* sp. 307-9 [11]. These findings have the potential to alleviate the antibiotic resistance crisis and demonstrate marine actinomycetes as a promising resource for lead compound discovery and development.

**Figure 1:**
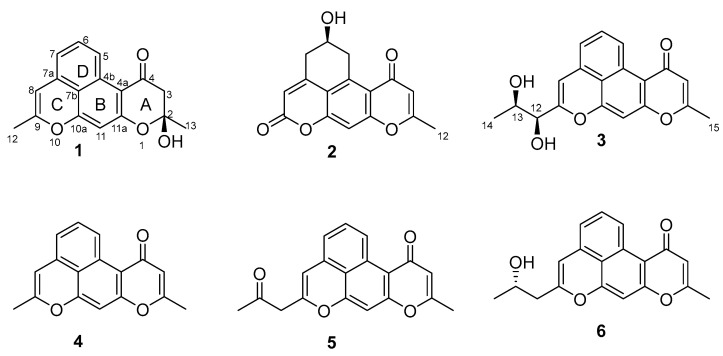
Structures of isolated compounds **1**–**6**.

In the ongoing process of our group’s research for natural products with excellent bioactivity from marine-derived microorganisms, a strain of *Streptomyces* sp. HDN150000 isolated from a marine sediment sample caught our attention. The OSMAC strategy (one strain–many compounds) was employed to cultivate *Streptomyces* sp. HDN150000, resulting in compounds with significantly different UV absorption profiles in M1 liquid media compared to other conditions. Under the guidance of HPLC-UV and LC-MS, six compounds with a 6/6/6/6 oxaphenalene pyranone skeleton, including three novels named naphpyrone I–K (**1**–**3**), were isolated and purified (Figure 1). Among them, compound **3** showed cytotoxic activities against cells L-02, K562, NCI-H446/EP, MDA-MB-231, and NCI-H446 with IC_50_ values of 5.13, 3.34, 2.50, 2.61, and 2.20 μM, respectively. Details of the isolation, structure elucidation, and bioactivities of these compounds are reported herein.

## 2. Results

**Figure 2:**
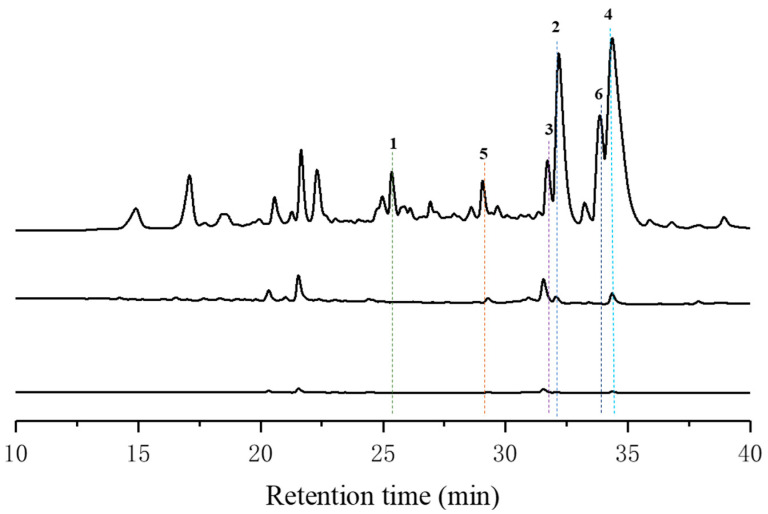
HPLC analysis of crude extracts of *Streptomyces* sp. HDN15000 in M1, TSB, and BF1 media.

*Streptomyces* sp. HDN15000 was isolated from a sediment sample collected from the South China Sea (125°28.550′ E, 29°1.618′ N). A comprehensive study of the metabolites produced by this *Streptomyces* species was carried out on various culture conditions. When culturing Streptomyces HDN150000 in M1 liquid medium, more differential peaks appeared (Figure 2).

To specifically identify the structures, we processed a larger-scale fermentation of this strain, and ethyl acetate extraction of the fermentation broth yielded 18.3 g crude extract. Subsequently, following HPLC results, the crude extracts were then separated stepwise using repeated silica gel column chromatography, Sephadex LH-20, and semipreparative HPLC to yield compounds **1**–**6** (Figure 1).

**Figure 3:**
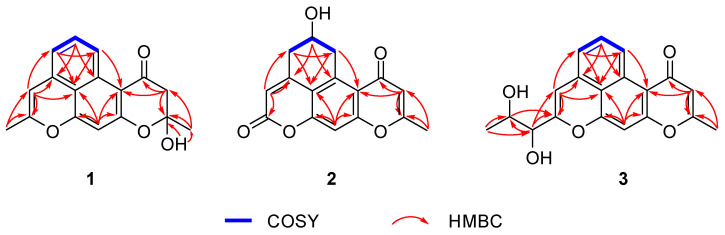
^1^H-^1^H COSY and key HMBC correlations of **1**–**3**.

Compound **1** was isolated as a white powder. The chemical formula was determined to be C_17_H_14_O_4_, supported by the cationic molecular ion peak at *m*/*z* 283.0962 [M + H]^+^ in the high-resolution electrospray ionization mass spectrometry (HRESIMS) (Figure S9), which signifies eleven degrees of unsaturation. The ^1^H NMR spectrum of **1** displayed two methyls (*δ*_H_ 2.13 and *δ*_H_ 1.62), one methylene (*δ*_H_ 3.05 and 2.68), five aromatic methines (*δ*_H_ 8.96, d, *J* = 8.8 Hz; *δ*_H_ 7.48, m; *δ*_H_ 6.88, d, *J* = 7.2 Hz; *δ*_H_ 6.22, s; *δ*_H_ 6.41, s) (Table 1). The ^13^C NMR spectrum combined with the HSQC spectrum reveals the existence of two methyls, one methylene, five protonated sp^2^ carbons, and nine non-protonated carbons (Table 1). The planar structure of **1** was established through a detailed analysis of its 2D NMR data. Firstly, the ^1^H-^1^H COSY correlations from H-5 (*δ*_H_ 8.98, d, *J* = 8.8 Hz) through H-6 (*δ*_H_ 7.48 m) to H-7 (*δ*_H_ 6.88, d, *J* = 7.2 Hz) suggest the existence of the 1,2,3-trisubstituted benzene ring (ring D). Further analysis of the key HMBC correlations from H-5 to C-7b/C-4a, from H-6 to C-7a/C-4b, and from H-7 to C-8/C-7b/C-5 identified the ring D and extended to the rings C and B. The HMBC correlations from H-12 to C-8/C-9, from H-8 to C-9/C-7a/C-7b, and from H-11 to C-7b/C-10a/C-4a/C-11a delineated rings C and B. Finally, the HMBC correlations from H-13 to C-2/C-3, from H-3 to C-4a/C-4/C-2, and from 2-OH (*δ*_H_ 7.12) to C-13/C-2 identified the ring A and its propagation to the ring B. Thus, the planar structure of compound **1** possessing 6/6/6/6 oxaphenalene pyranone was determined (Figure 3).

**Figure 4:**
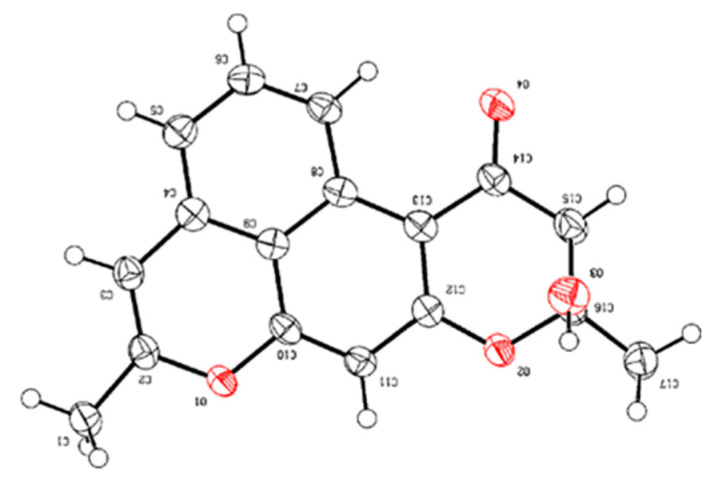
ORTEP drawing for crystal structure of **1**.

The absolute configuration of compound **1** was confirmed as 2*S* supported by the X-ray diffraction (Figure 4).

**Figure 5:**
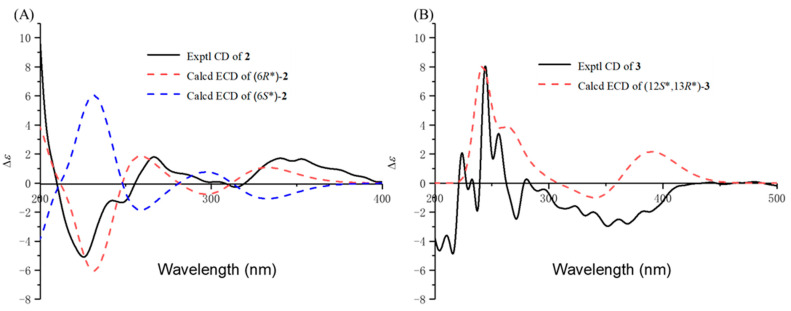
(**A**) Calculated and experimental ECD spectra of **2**. (**B**) Calculated and experimental ECD spectra of **3**.

Compound **2** was obtained as a white solid powder. The molecular formula C_16_H_12_O_5_ deduced by HR-ESIMS, suggests 11 degrees of unsaturation. ^1^H and ^13^C spectra reveal that compound **2** contains one methyl, two methylenes, one sp^3^ methine, three sp^2^ methine, and nine non-protonated carbons (including two carbonyl and seven aromatic quaternary carbons). Detailed 2D NMR analysis showed that compound **2** has a similar 6/6/6/6 system with compound **1** except that the dehydration of C-2 and C-3 of the ring A to form a double bond, which can be determined by the HMBC correlations from H-12 (*δ*_H_ 2.34) to C-2 (*δ*_C_ 165.3) and C-3 (*δ*_C_ 111.3). The C-5 (*δ*_C_ 36.7), C-6 (*δ*_C_ 63.2), and C-7 (*δ*_C_ 36.7) of the ring D changed from sp^2^ to sp^3^ carbon signals determined from the ^1^H-^1^H COSY correlations of H-5/H-6/H-7. Further consideration of the C-6 as the methenyl carbon signal suggests the presence of hydroxyl substitution [12]. Another difference lies in the low-field chemical shift of C-9 (*δ*_C_ 159.1) in combination with the HR-ESIMS data at *m/z* 285.0755 [M + H]^+^, identifying C-9 as a carbonyl carbon [13,14]. Thus, the planar structure of **2** was determined (Figure 3). To determine the absolute configuration of **2**, ECD calculations of the possible configurations (6S*)-**2** and (6R*)-**2** were performed. Finally, the good agreement between the experimental and calculated ECD curves evidenced that the absolute configuration of **2** is 6*R* (Figure 5A).

Compound **3** was separated as a white amorphous solid powder. The molecular formula of C_19_H_16_O_5_ is established by the HRESIMS data (*m*/*z* 325.1073 [M + H]^+^, calcd. 325.1071). The ^1^H NMR spectrum of **3** showed two methyls (*δ*_H_ 1.13 and *δ*_H_ 2.53), two sp^3^ methines (*δ*_H_ 4.00 and *δ*_H_ 3.91), six sp^2^ aromatic methines (*δ*_H_ 6.22; *δ*_H_ 9.48; *δ*_H_ 7.60; *δ*_H_ 7.16; *δ*_H_ 6.43; *δ*_H_ 6.88) (Table 1). The ^13^C NMR spectrum reveals the existence of two methyls, eight methines, and nine non-protonated carbons (including a carbonyl and eight non-protonated sp^2^ carbons) (Table 1). 2D NMR showed that compound **3** has a 6/6/6/6/6 ring system structure similar to compound **1,** except that C-2 (*δ*_C_ 163.4) and C-3 (*δ*_C_ 112.2) are dehydrated to form a double bond, in addition the substitution at C-9 (*δ*_C_ 156.3) with a propylene glycol side chain can be further determined by the HMBC correlations from H-14 (*δ*_H_ 1.13) to C-13 (*δ*_C_ 67.4) and C 12 (*δ*_C_ 74.1), from H-13 (*δ*_H_ 3.91) to C-9, and from H-12 (*δ*_H_ 4.00) to C-8 (δC 104.2), C-9, and C-13. At this point, the planar structure of compound **3** was determined.

Compound **3** has four possible configurations (12S*,13R*)-**3**, (12S*,13S*)-**3**, (12R*,13R*)-**3**, and (12R*,13S*)-**3**. To determine the relative structure of **3**, the theoretical NMR calculations at PCM(DMSO)-mPW1PW91/6-311+G(d,p)//B3LYP/6-31G(d)-GD3BJ level and DP4+ probability analyses were employed on two possible configurations (12S*,13R*)-**3** and (12S*,13S*)-**3**, and the calculations messages suggested that (12S*,13R*)-**3** was the correct relative structure (Table S1). Subsequent ECD calculations were performed and compared to the experimental ECD curves to determine the absolute conformation of **3** as 12*S*,13*R* (Figure 5B).

Based on the comparison of NMR and MS data with those reported in the literature, the known compounds isolated in this study were identified as naphpyrone D (**4**), naphpyrone B (**5**), and naphpyrone C (**6**) [15].

All new compounds were evaluated for cytotoxicity against L-02, MDA-MB-231, K562, ASPC-1, NCI-H446, and NCI-H446/EP cell lines in vitro. Adriamycin was used as the positive control. As a result, compound **3** showed cytotoxic activity against cells L-02, K562, NCI-H446/EP, MDA-MB-231, and NCI-H446 with IC_50_ values of 5.13, 3.34, 2.50, 2.61, and 2.20 μM, respectively (Table S2–S6).

## 3. Materials and Methods

### 3.1. General Experimental Procedures

*Streptomyces* sp. HDN150000 genomes were acquired using methods previously described in the literature [16].

An Agilent DD2-500 spectrometer was used to obtain NMR spectra, with tetramethylsilane (TMS) as the internal standard. UV-vis spectra were recorded on the UFLC system (Shimadzu, Tokyo, Japan). HRESIMS spectra were measured on a Thermo Scientific LTQ Orbitrap XL mass spectrometer (Thermo Fisher Scientific, Bremen, Germany). Specific rotations were obtained on a JASCO P-1020 digital polarimeter. The ECD spectra were obtained using a JASCO J-715 spectropolarimeter (JASCO, Tokyo, Japan). Column chromatography (CC) was performed using silica gel (300–400 mesh, Qingdao Marine Chemical Ltd., Qingdao, China), SiliaSphere C18 (Octadecylsilyl, ODS) monomeric (SiliCycle Inc., Québec City, Canada, 50 μm, 120 A), and Sephadex LH-20 (Amersham Biosciences, Buckinghamshire, UK). MPLC was performed on a Waters 1526. HPLC spectra were collected with an ODS column (YMC-Pack ODS-A, 10 × 250 mm, 5 μm, 3 mL min^−1^, YMC Co., Ltd., Kyoto, Japan).

### 3.2. Materials and Culture Conditions

*Streptomyces* sp. HDN155000 was isolated from a marine sediment sample collected from the South China Sea at coordinates 125°28.550′ E and 29°1.618′ N. The strain was identified through genome sequencing and submitted to GenBank (No. OP001709) [17]. Wild type and their mutant strains were cultivated for 8 days at 28 °C on MS plates containing 2% soya bean flour, 2% mannitol, and 1.8% agar. For genome extraction, the strain was inoculated into 250 mL Erlenmeyer flasks with 50 mL of BF1 liquid medium (1% glucose, 1% tryptone, 2% starch, 0.5% yeast extract, and 0.5% CaCO_3_) for 2 days.

### 3.3. Fermentation and LC/LC-MS Analysis

For small-scale analysis, all actinobacteria were cultured for 8 days at 28 °C in M1 medium (4 g/L yeast extract, 2 g/L peptone, 10 g/L starch), TSB medium (1.7% tryptone, 0.3% peptone, 0.25% glucose, 0.25% KH2PO4, and 0.3% NaCl) and BF1 medium after which the culture products were collected and extracted by ethyl acetate at least three times. The organic phase from the three extractions was evaporated, and the residue was redissolved in 200 μL MeOH. Then, 50 μL of dissolved extract was injected for high-performance liquid chromatography–photodiode array detection–mass spectrometry (HPLC-DAD-MS) analysis (C18 column, Shimadzu, 4.6 mm × 150 mm, 5 μm, 1 mL/min); the samples were separated on a linear gradient of 5–100% acetonitrile (MeOH) in water (0.1% trifluoroacetic acid) for 50 min at a flow rate of 1 mL/min, and HPLC analysis revealed a significant change in metabolite production in the mutant strain.

For compound separation, the selected strain was incubated in M1 medium at 28 °C for 2 days as a seed solution. Then, cultured seed solutions were transferred to 500 mL Erlenmeyer flasks containing 100 mL of M1 medium at 2% inoculum (total 18 L) for further fermentation. The broth was extracted three times with ethyl acetate to provide a total of 55 L of extract solution. The organic phase was evaporated under reduced pressure to afford a crude residue (18.3 g).

### 3.4. Extraction, Isolation, and Purification

To identify secondary metabolites, under the guidance of HPLC analysis, the crude extracts were separated with a step gradient elution of MeOH-H_2_O, providing eight subfractions (Fr.1–Fr.8, 30% to 100%). The compounds are mainly concentrated in Fr.3–Fr.5. Fr.3 was purified by semi-preparative HPLC (41:59 MeOH-H_2_O, 3 mL/min) to generate compound **1** (5.4 mg, *t*_R_ = 21.3 min). Fr.3 was purified by a semi-preparative C18 HPLC column (55:45 MeOH-H_2_O), producing compound **3** (3.8 mg, *t*_R_ = 14.7 min) and Fr.4.1. This subfraction was separated with preparative C18 HPLC column (50:50 MeOH-H_2_O) to yield compounds **2** (4.3 mg, *t*_R_ = 14.7 min) and **5** (3.4 mg, *t*_R_ = 18.5 min). Fr.5 was applied to a Sephadex LH-20 column and eluted with methanol, obtaining compound **4** (8.2 mg). Fr.5 was purified through semipreparative HPLC (66:34 MeOH-H_2_O, 3 mL/min) to produce compound **6** (9.0 mg, *t*_R_ = 13.3 min).

Naphpyrone I (**1**): white powder, ${\left[\alpha \right]}_{D}^{25}$ –32.6 (*c* 0.03, CH_3_OH); UV (DAD) *λ*_max_ 210 nm, 226 nm, 242 nm, 254 nm, 277 nm, 242 nm, and 399 nm; CD (MeOH); *λ*_max_ (∆ε) 210 (–12.46), 218 (+3.21), 226 (–1.78), 232 (–0.36), 240 (–2.09), and 263 (+2.35); ^1^H and ^13^C NMR data, see Table 1; positive ion HRESIMS *m/z* 283.0962 [M + H]^+^ (calcd. for C_17_H_15_O_4_^+^, 283.0965).

Naphpyrone J (**2**): white solid powder, ${\left[\alpha \right]}_{D}^{25}$ 19.5 (*c* 0.03, CH_3_OH); UV (DAD) *λ*_max_ 210 nm, 225 nm, 258 nm, 263 nm, 269 nm, 274 nm, 279 nm, and 329 nm; CD (MeOH); *λ*_max_ (∆ε) 225 (–5.08), 266 (+1.81), 315 (–0.17), and 353 (+1.66); ^1^H and ^13^C NMR data, see Table 1; positive ion HRESIMS *m/z* 285.0755 [M + H]^+^ (calcd. for C_16_H_13_O_5_^+^, 285.0757).

Naphpyrone K (**3**): white amorphous solid powder, ${\left[\alpha \right]}_{D}^{25}$ –20.3 (*c* 0.03, CH_3_OH); UV (DAD) *λ*_max_ 238 nm, 242 nm, 258 nm, 270 nm, 274 nm, and 274 nm; CD (MeOH); *λ*_max_ (∆ε) 224 (+2.16), 244 (+8.21), 251 (+1.66), 356 (+3.36), and 369 (–2.82); ^1^H and ^13^C NMR data, see Table 1; positive ion HRESIMS *m/z* 325.1073 [M + H]^+^ (calcd. for C_19_H_17_O_7_^+^, 325.1071).

### 3.5. X-Ray Crystallographic Analysis of Naphpyrone I

Naphpyrone I (**1**) was analyzed by X-ray diffraction on Cu Kα radiation. Compound **1** was concentrated in vacuo and redissolved in 1.5 mL CH_3_OH that was transferred in a 2 mL clear screw autosampler vial. The lid with holes was then closely screwed on the vial, and white needle-like crystals were obtained after seven nights at room temperature.

Crystal Data: C_17_H_14_O_4_ (M =282.28 g/mol): trigonal, space group R-3 (no. 148), *a* = 36.3042(8) Å, *c* = 5.5043(2) Å, *V* = 6282.7(4) Å^3^, *Z* = 18, *T* = 120.00(10) K, μ (Cu Kα) = 0.789 mm^−1^, *Dcalc* = 1.343 g/cm^3^, 7569 reflections measured (4.868° ≤ 2Θ ≤ 145.992°), 2693 unique (*R*_int_ = 0.0599, R_sigma_ = 0.0589) which were used in all calculations. The final *R*_1_ was 0.0530 (I > 2σ(I)) and *wR*_2_ was 0.1540 (all data).

The crystallographic data have been deposited in the Cambridge Crystallographic Data Centre as CCDC 2434219.

### 3.6. NMR and ECD Calculations

Conformational searches were conducted using Spartan′14, based on the MMFF force fields [18]. Compounds **1**, **2**, and **3** were further optimized with DFT calculations at the B3LYP/6-31G(d)-GD3BJ level, utilizing the Gaussian 16 program package [19]. Gauge-independent atomic orbital (GIAO) calculations of ^13^C NMR of conformers were accomplished by DFT at the mPW1PW91/6-311+G(d,p) level with the PCM model in DMSO. The calculated NMR data of these conformers were averaged according to the Boltzmann distribution theory and their relative Gibbs free energy. The ^13^C NMR chemical shifts for TMS were also calculated by the same procedures and used as the reference. After calculation, the experimental and calculated data were evaluated by the improved probability DP4+ method [20]. The ECD was calculated using time-dependent density functional theory (TDDFT) at a B3LYP/6-31+G(d) level in methanol with the IEFPCM model [21]. The calculated ECD curves were all generated using the SpecDis 1.71 program package, and the calculated ECD data of all conformers were Boltzmann averaged using Gibbs free energy [22].

### 3.7. Cytotoxicity Assay

The cytotoxic assay involved six human cancer cell lines: K562 (using the MTT method), L-02, MDA-MB-231, ASPC-1, NCI-H446, and NCI-H446/EP (using the SRB method). Adriamycin (ADM) was used as a positive control. The detailed procedures for biological testing were conducted as previously stated [23]. The cancer cell lines were purchased from the National Collection of Authenticated Cell Cultures of China (Shanghai).

## 4. Conclusions

Marine actinobacteria can produce structurally diverse secondary metabolites with unique biological activities, making them a new resource for the discovery of antibiotics. Six compounds containing a 6/6/6/6 oxaphenalene pyranone skeleton were isolated and determined from *Streptomyces* sp. HDN150000, which was isolated from a marine sediment sample collected from the South China Sea. Among them, compound **3** showed cytotoxic activity against cells L-02, K562, NCI-H446/EP, MDA-MB-231, and NCI-H446, providing an alternative lead compound for medical study. Our study highlights the potential of mining secondary metabolites from marine actinobacteria for screening and utilization of therapeutic molecules.

## Figures and Tables

**Table 1:** ^1^H (500 MH_Z_) and ^13^C (125 MH_Z_) spectroscopic data for **1**–**3** in DMSO-*d*_6_.

No	1		2		3	
	*δ*_C_ Type	*δ*_H_ (*J* in Hz)	*δ*_C_ Type	*δ*_H_ (*J* in Hz)	*δ*_C_ type	*δ*_H_ (*J* in Hz)
2	101.5 qC		165.3 qC		163.4 qC	
3	49.3 CH_2_	3.05 day (15.9)	111.3 CH	6.17 s	112.2 CH	6.22 day (1.0)
		2.68 day (15.9)				
4	190.6 qC		178.6 qC		177.7 qC	
4a	106.4 qC		117.7 qC		110.5 qC	
4b	132.6 qC		140.2 qC		131.6 qC	
5	121.2 CH	8.96 day (8.8)	36.7 CH_2_	3.69 m	122.7 CH	9.48 day (8.8, 1.0)
				3.64 m		
6	131.8 CH	7.48 m	63.2 CH	4.15 m	131.3 CH	7.60 m
7	115.5 CH	6.88 day (7.2)	36.7 CH	2.97 m	118.0 CH	7.16 day (7.3, 1.0)
				2.80 m		
7a	129.4 qC		153.7 qC		129.2 qC	
7b	117.2 qC		114.2 qC		119.7 qC	
8	104.4 CH	6.22 s	111.9 CH	6.32 s	104.2 CH	6.43 brs
9	152.3 qC		159.1 qC		156.3 qC	
10a	158.6 qC		155.2 qC		156.9 qC	
11	98.8 CH	6.41 s	103.1 CH	7.41 s	97.4 CH	6.88 s
11a	162.9 qC		158.8 qC		159.4 qC	
12	18.7 CH_3_	2.13 s	19.5 CH_3_	2.34 s	74.1 CH	4.00 day (4.7)
13	27.3 CH_3_	1.62 s			67.4 CH	3.91 m
14					19.3 CH_3_	1.13 day (6.4)
15					19.2 CH_3_	2.35 s
2-OH		7.12 s				

## Data Availability

The data given in this research are available in this article and the Supplementary Materials.

## Supplementary Materials

The following supporting information can be downloaded at: https://www.mdpi.com/article/10.3390/md23050188/s1, Figure S1: Separation process of *Streptomyces*. sp. HDN150000 mutant strain extract in M1; Figure S2: UV absorption of compounds **1**-**6**. Figure S3: Two relative configurations **3a** and **3b**; Figure S4: Calculated and experimental ECD spectra of **3**; Figures S5–S10: NMR and HRESIMS spectra of compound **1**; Figures S11–S16: NMR and HRESIMS spectra of compound **2**; Figures S17–S22: NMR and HRESIMS spectra of compound **3**; Table S1: DP4+ analysis results of **3a** (Isomer 1) and **3b** (Isomer 2); Tables S2–S6: Cytotoxicity assay of novel compound **3**.
