# Supplementary material for: Polyketides with a 6/6/6/6 Oxaphenalene Pyranone Skeleton from Marine-Derived Streptomyces sp. HDN150000
Source: Mar Drugs. 2025 Apr 27;23(5):188. doi: 10.3390/md23050188 (PMC12112823; doi:10.3390/md23050188)
Supplement: Supplementary file 1 [file marinedrugs-23-00188-s001.zip › marinedrugs-3582359-supplementary.pdf]

Polyketides with a 6/6/6/6 oxaphenalene Pyranone Skeleton from Marine-derived *Streptomyces* sp. HDN150000

Xiaoting Zhang, Falei Zhang, Wenxue Wang, Xingtao Ren Tianjiao Zhu, Qian Che, Dehai Li, Guojan Zhang

Contents

Section S1. Separation process of the *Streptomyces*. sp. HDN150000..................2

Section S2. Calculational details for **3**..2

Section S3. Compound **3** was evaluated for cytotoxicity. 4

Section S4. NMR and HRESIMS spectra of **1** 6

Section S5. NMR and HRESIMS spectra of **2** 9

Section S6. NMR and HRESIMS spectra of **3** 12

Section S1. Separation process of the *Streptomyces*. sp. HDN150000

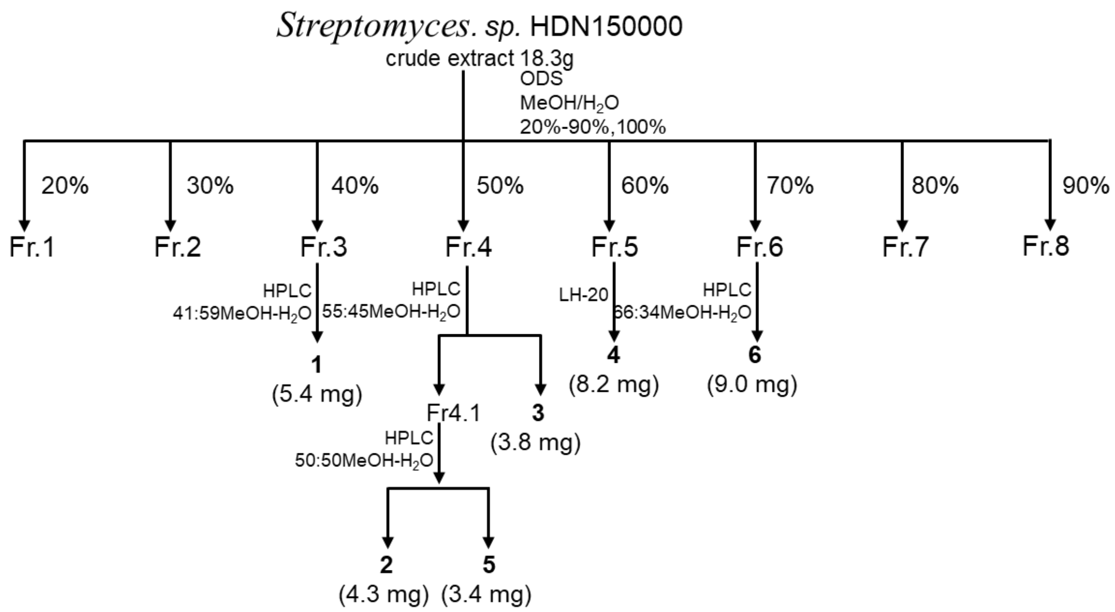

Figure S1. Separation process of the *Streptomyces*. sp. HDN150000 strain extract in M1

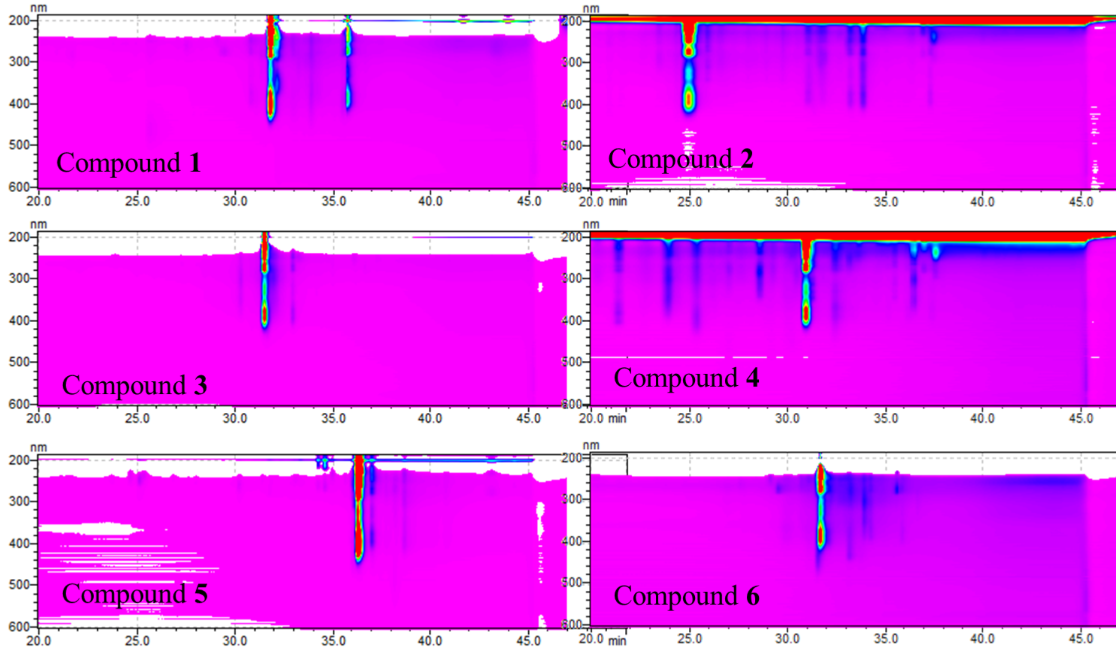

Figure S2. UV absorption of compounds **1-6**

Section S2. Calculational details for 3

There are four possible configurations of the compound **3**. Two relative configurations, **3a** and **3b**, were calculated at PCM(DMSO)-mPW1PW91/6-311+G(d,p)//B3LYP/6-31G(d)-GD3BJ level and then DP4+ probability analyses suggested that (12*S**,13*R**)-**3a** was the correct relative structure, followed by ECD

calculations at the IEFPCM(methanol)-B3LYP/6-31+G(d)//B3LYP/6-31G(d)-GD3BJ level to determine the absolute configuration of **3** as 12*S*,13*R*.

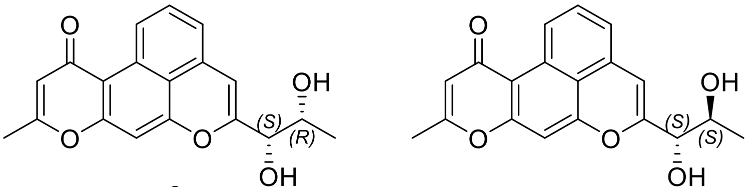

3a **3b**

Figure S3. Two relative configurations **3a** and **3b**

Table S1. DP4+ analysis results of **3a** (Isomer 1) and **3b** (Isomer 2)

Functional	Solvent?	Basis Set	Type of Data
mPW1PW91	PCM	6-31+G(d, p)	Shielding Tensors

		DP4+	83. 16%	16. 84%	–	–	–
Nuclei	sp2?	xperimenta	Isomer 1	Isomer 2	Isomer 3	Isomer 4	Isomer 5
C	x	104. 2	76. 6	76. 3			
C	x	129. 2	52. 9	52. 8			
C	x	119. 7	63. 0	63. 0			
C	x	156. 9	23. 7	23. 9			
C	x	156. 3	24. 4	24. 8			
C		74. 1	110. 8	112. 0			
C	x	118	64. 6	64. 7			
C	x	131. 8	50. 1	50. 1			
C	x	122. 7	58. 9	59. 0			
C	x	131. 6	49. 4	49. 4			
C	x	110. 5	71. 4	71. 4			
C	x	159. 4	21. 41	21. 42			
C	x	97. 4	85. 18	85. 26			
C	x	177. 7	3. 99	3. 98			
C	x	112. 2	68. 91	68. 91			
C	x	163. 4	15. 49	15. 51			
C		19. 3	165. 69	165. 67			
C		67. 4	114. 58	113. 88			
C		19. 2	168. 35	168. 68			

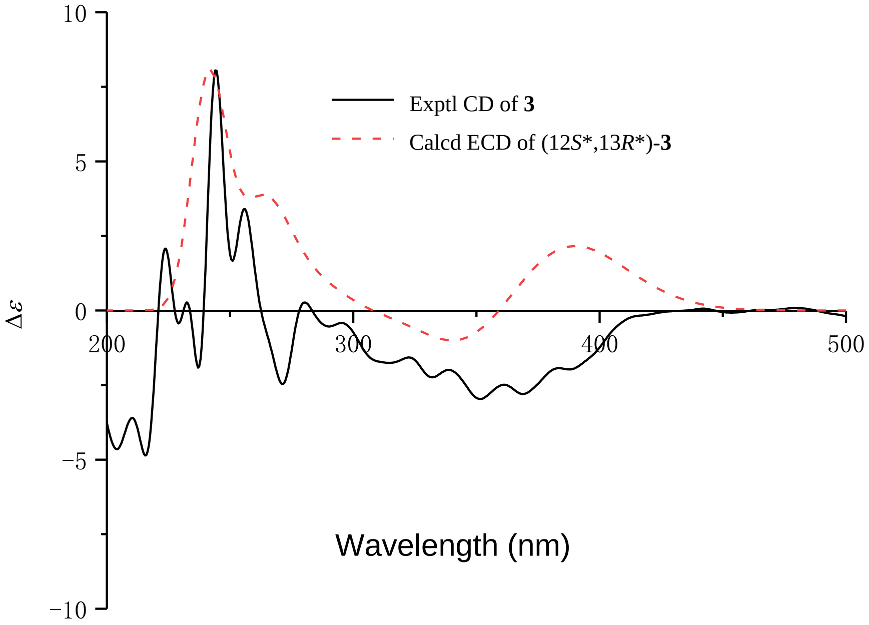

Figure S4. Calculated and experimental ECD spectra of **3**.

Section S3. Compound 3 was evaluated for cytotoxicity.

Table S2. Cytotoxicity assay of novel compound **3** against L-02 cell line

	Concentration (μM)	OD	inhibition rate %	IC_{50} (μM)
blank group		1.22±0.01		
control group		1.17±0.06		
adriamycin	1	0.09±0.01	92.56	0.44
**3**	30	0.21±0.02	83.10	5.13
	15	0.30±0.03	75.27	
	7.5	0.39±0.02	68.15	
	3.75	0.47±0.03	61.76	
	1.875	0.84±0.03	31.01	
	0.9375	1.15±0.06	5.15	

Table S3. Cytotoxicity assay of novel compound **3** against K562 cell line

	Concentration (μM)	OD	inhibition rate %	IC_{50} (μM)
blank group		1.66±0.02		
control group		1.67±0.04		
adriamycin	1	0.14±0.01	100.00	0.24
**3**	30	0.33±0.02	87.65	3.34
	15	0.56±0.01	72.36	
	7.5	0.60±0.03	69.54	
	3.75	0.72±0.01	61.89	
	1.875	0.99±0.04	44.15	
	0.9375	1.39±0.04	17.64	

Table S4. Cytotoxicity assay of novel compound **3** against NCI-H446/EP cell line

	Concentration (μM)	OD	inhibition rate %	IC_{50} (μM)
blank group		1.35±0.01		
control group		1.35±0.03		
adriamycin	1	0.75±0.04	44.21	1.02
**3**	30	0.15±0.01	88.90	2.50
	15	0.31±0.01	76.93	
	7.5	0.42±0.01	68.61	
	3.75	0.45±0.02	66.95	
	1.875	0.58±0.03	57.23	
	0.9375	1.08±0.04	20.15	

Table S5. Cytotoxicity assay of novel compound **3** against MDA-MB-231 cell line

	Concentration (μM)	OD	inhibition rate %	IC_{50} (μM)
blank group		1.58±0.01		
control group		1.55±0.01		

adriamycin	1	0.11±0.01	93.22	0.31
	30	0.16±0.01	90.07	
	15	0.12±0.01	92.33	
	7.5	0.15±0.03	90.71	
**3**	3.75	0.29±0.04	81.88	2.61
	1.875	1.19±0.05	24.59	
	0.9375	1.62±0.01	-2.78	

Table S6. Cytotoxicity assay of novel compound **3** against NCI-H446 cell line

	Concentration (μM)	OD	inhibition rate %	IC_{50} (μM)
blank group		0.30±0.03		
control group		0.29±0.02		
adriamycin	1	0.06±0.01	80.13	0.63
	30	0.07±0.01	76.77	2.20
	15	0.08±0.03	74.75	
	7.5	0.08±0.03	72.90	
**3**	3.75	0.07±0.01	77.61	
	1.875	0.13±0.01	57.58	
	0.9375	0.28±0.01	4.71	

Section S4. NMR and HRESIMS spectra of 1

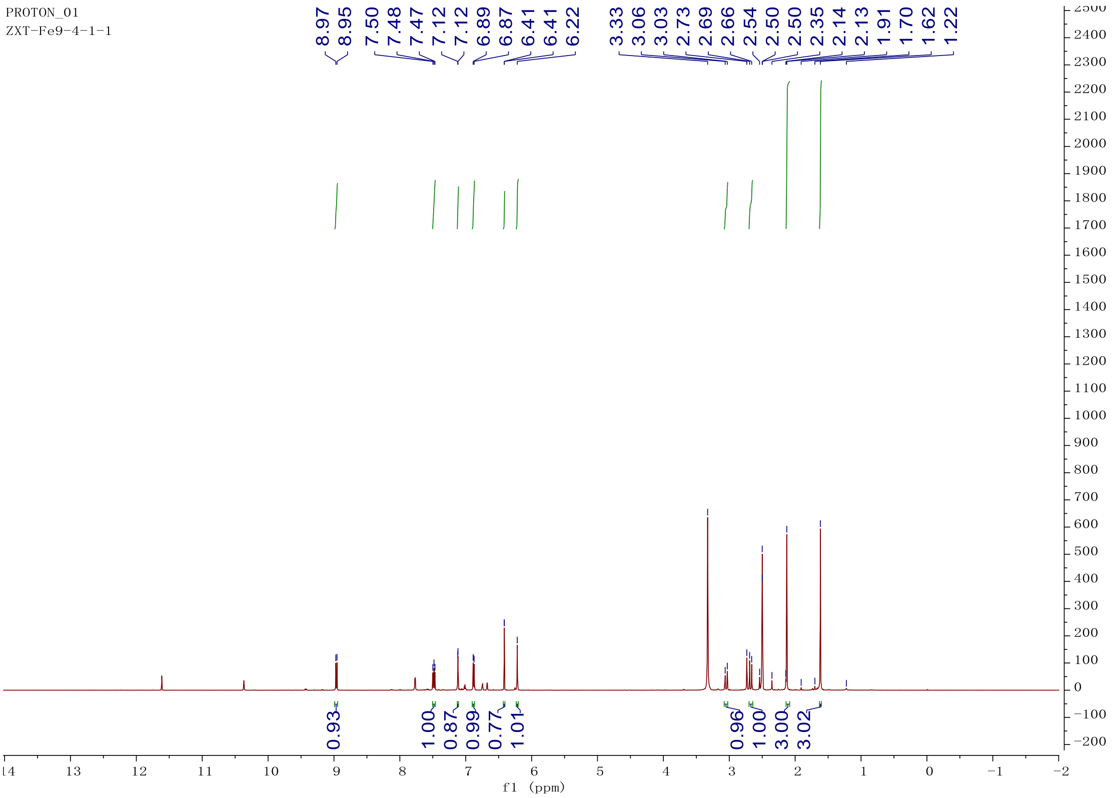

Figure S5 ^{1}H NMR spectrum (500 MHz) of naphpyrone I (**1**) in DMSO-d_6

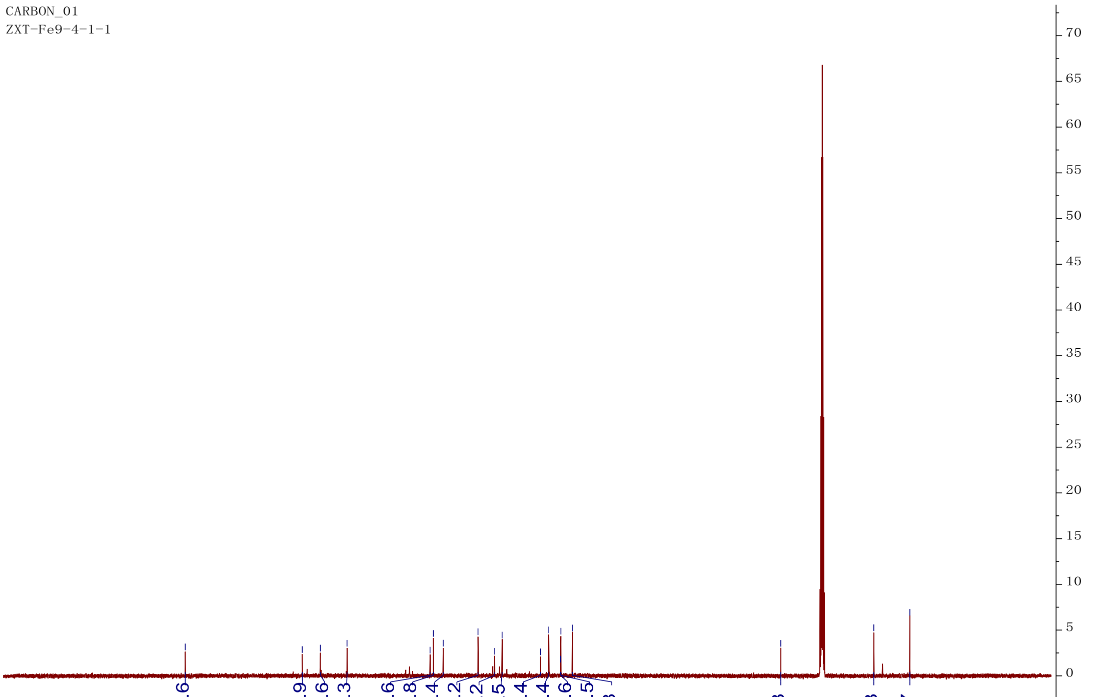

Figure S6 ^{13}C NMR spectrum (125 MHz) of naphpyrone I (**1**) in DMSO-d_6

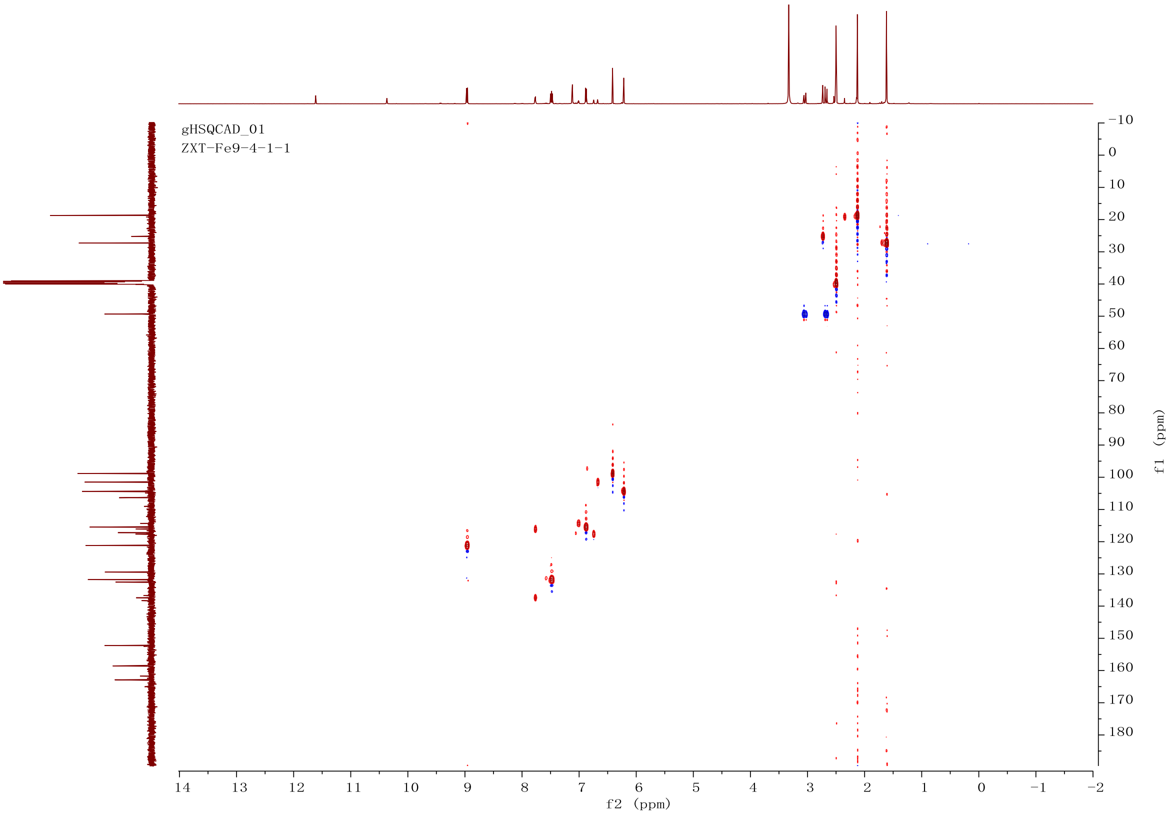

Figure S7 HSQC spectrum of naphpyrone I (**1**) in DMSO-d_6

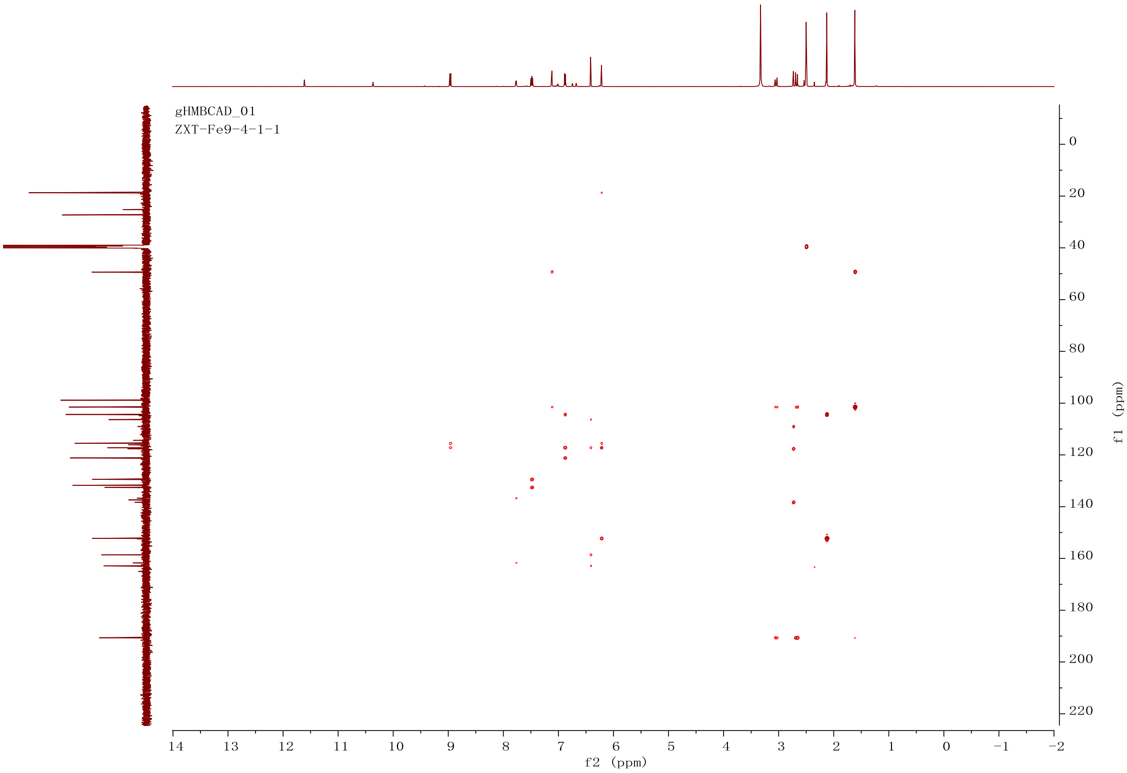

Figure S8 HMBC spectrum of naphpyrone I (**1**) in DMSO-d_6

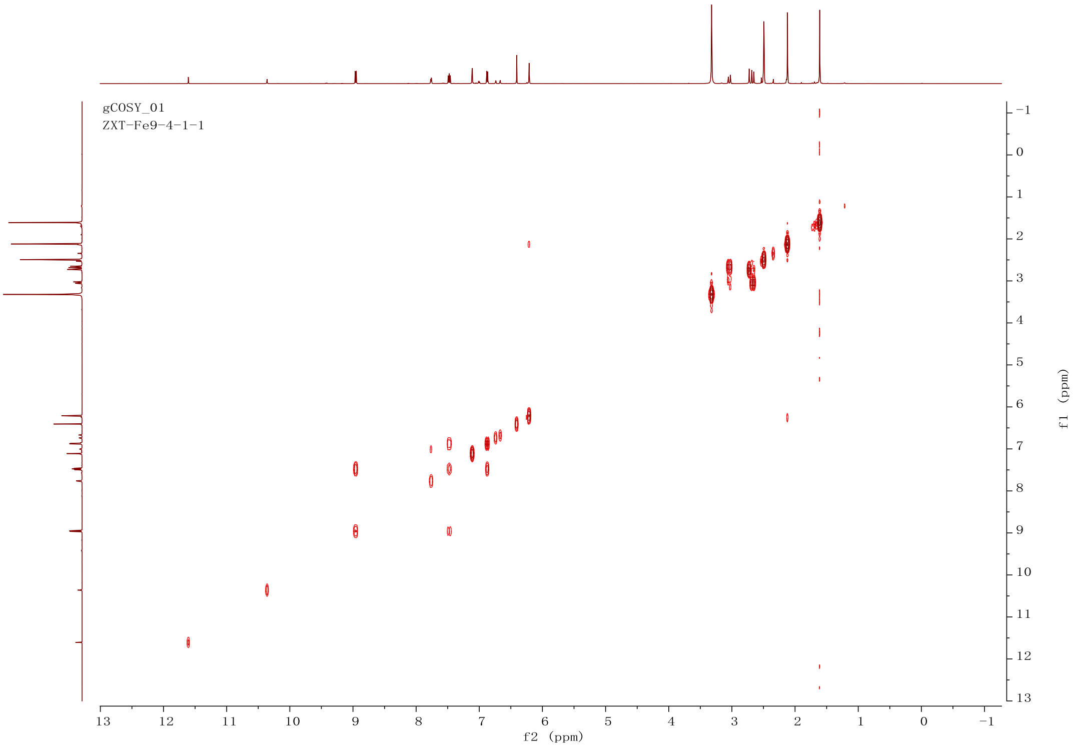

Figure S9 ^{1}H-^{1}H COSY spectrum of naphpyrone I (**1**) in DMSO-d_6

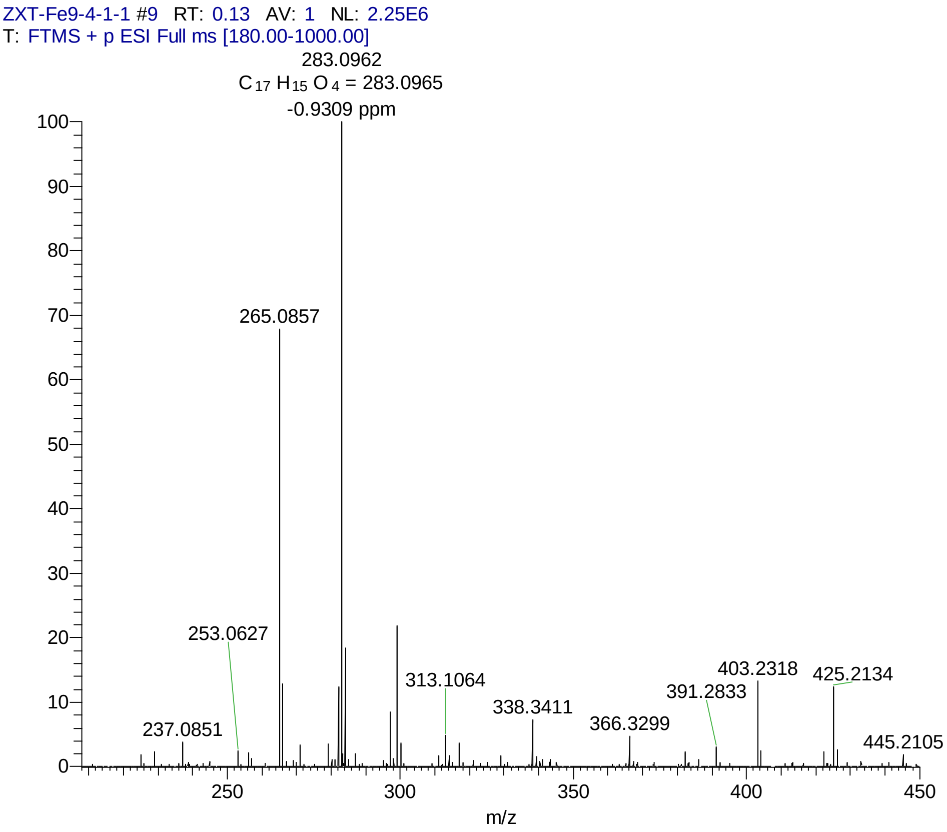

Figure S10 HRESIMS spectrum of naphpyrone I (**1**)

Section S5. NMR and HRESIMS spectra of 2

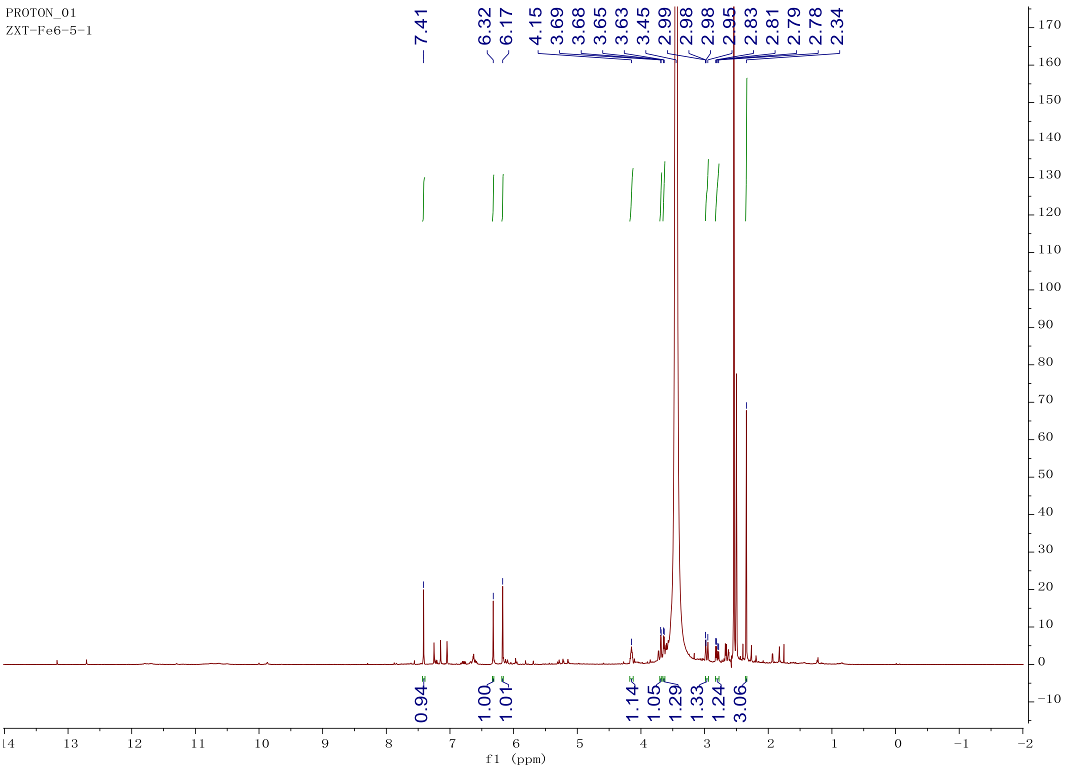

Figure S11 ^{1}H NMR spectrum (500 MHz) of naphpyrone J (**2**) in DMSO-d_6

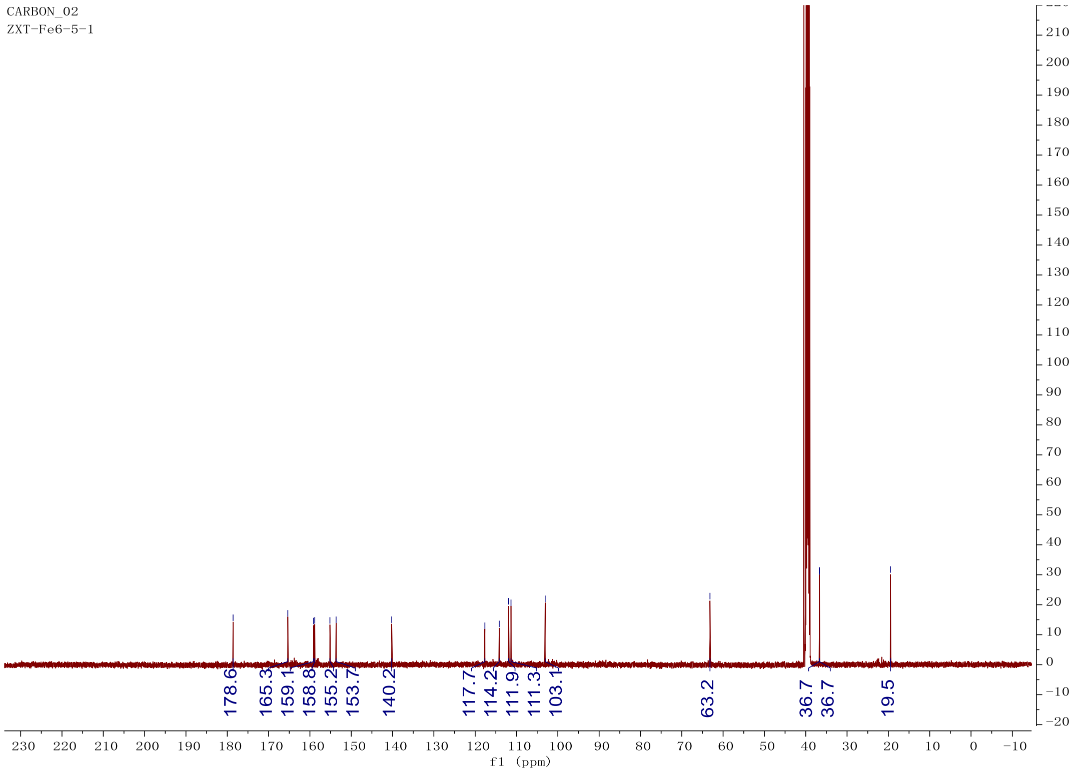

Figure S12 ^{13}C NMR spectrum (125 MHz) of naphpyrone J (**2**) in DMSO-d_6

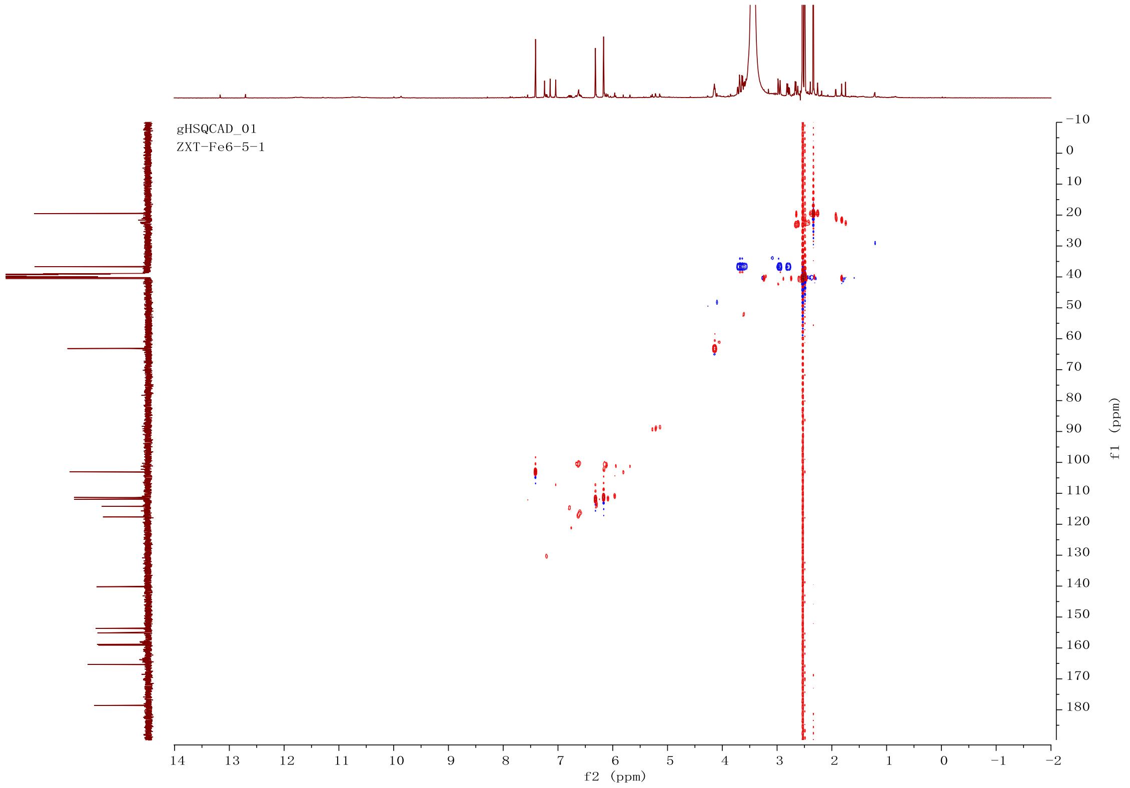

Figure S13 HSQC spectrum of naphpyrone J (**2**) in DMSO-d_6

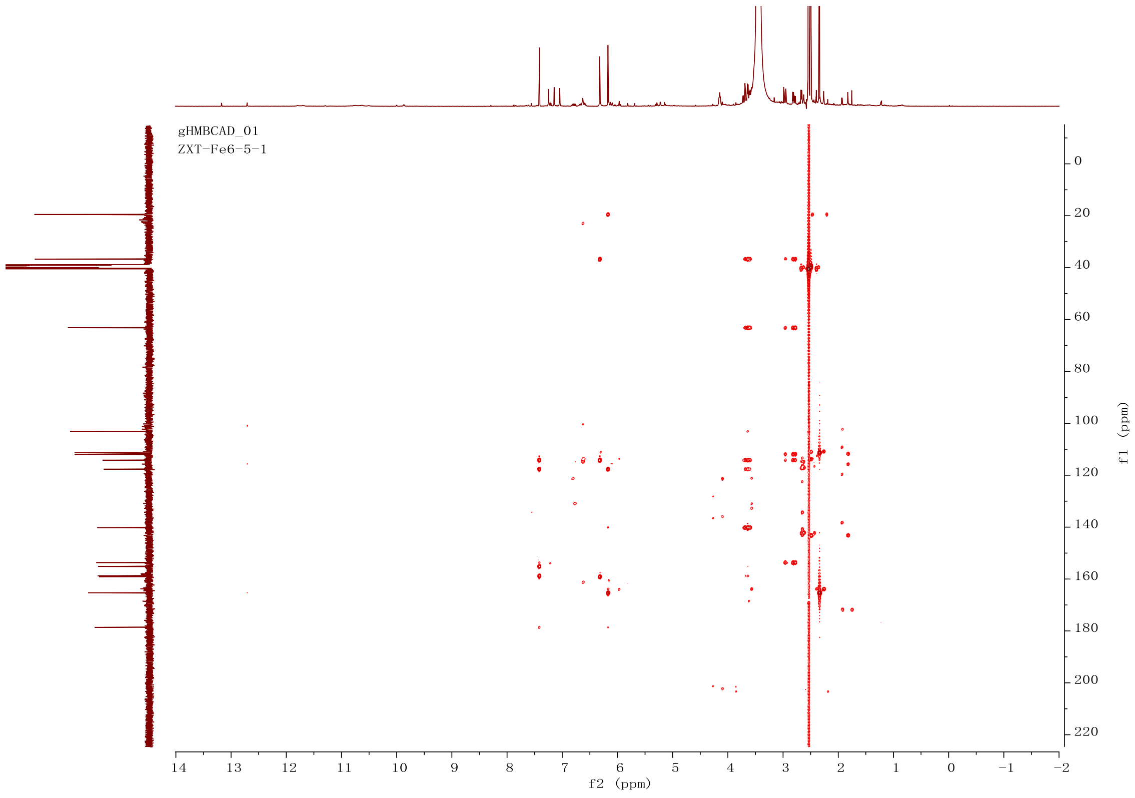

Figure S14 HMBC spectrum of naphpyrone J (**2**) in DMSO-d_6

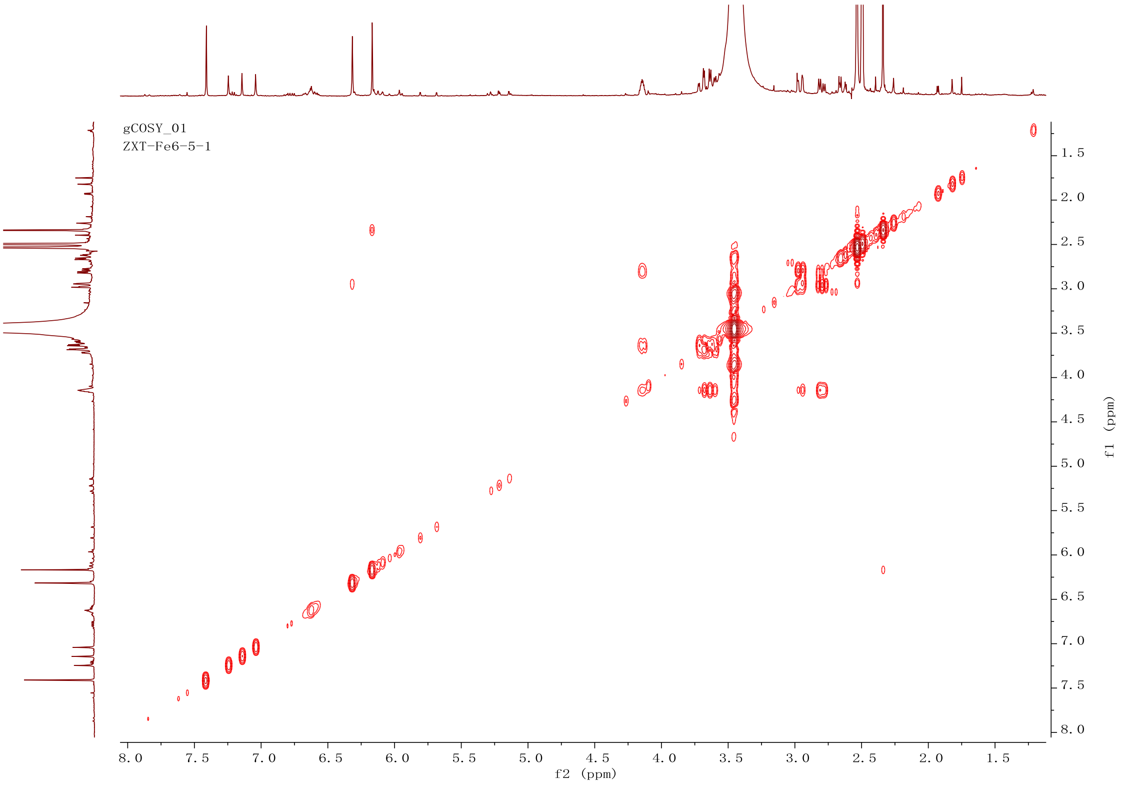

Figure S15 ^{1}H-^{1}H COSY spectrum of naphpyrone J (**2**) in DMSO-d_6

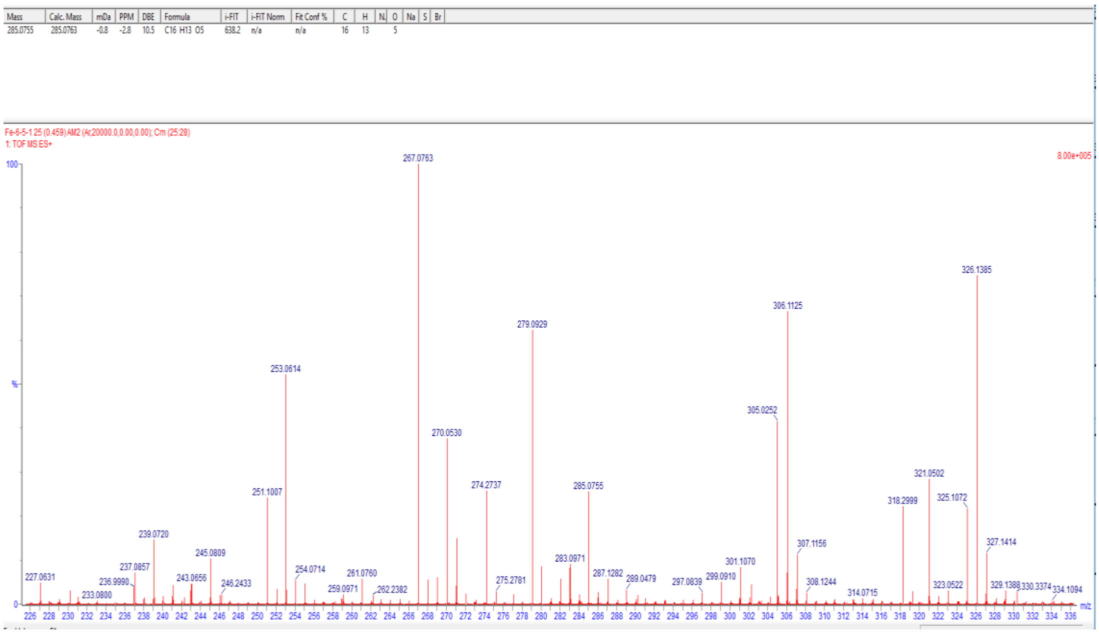

Figure S16 HRESIMS spectrum of naphpyrone J (**2**)

Section S6. NMR and HRESIMS spectra of 3

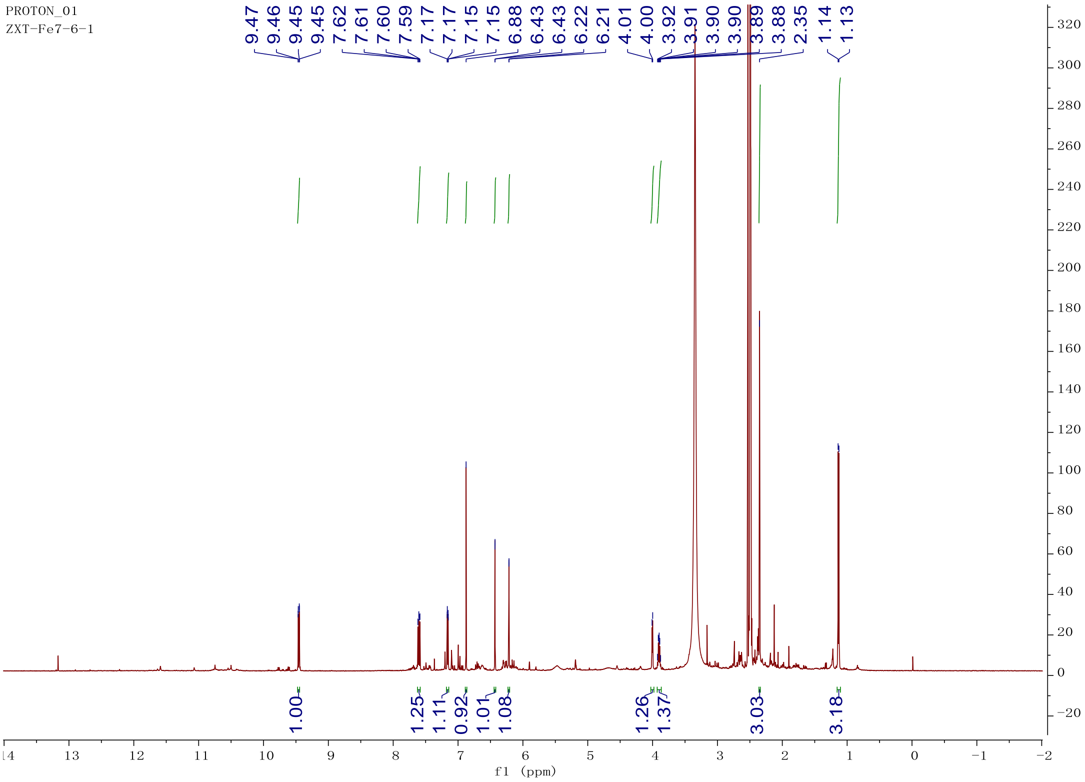

Figure S17 ^{1}H NMR spectrum (500 MHz) of naphpyrone K (**3**) in DMSO-d_6

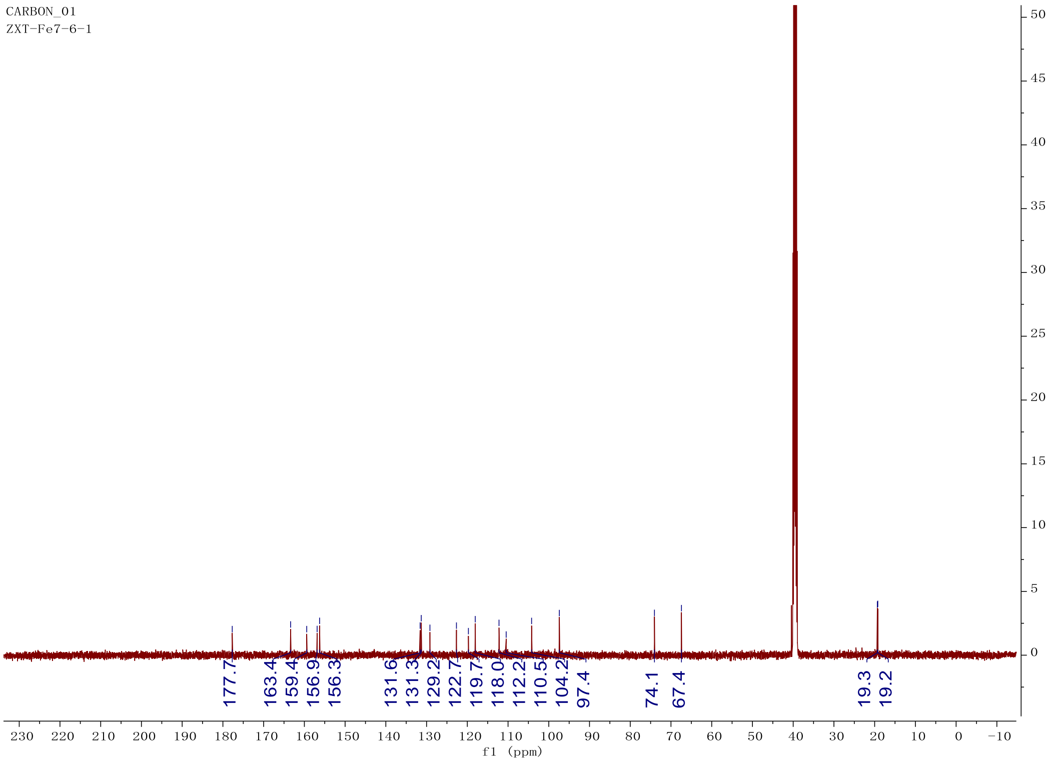

Figure S18 ^{13}C NMR spectrum (125 MHz) of naphpyrone K (**3**) in DMSO-d_6

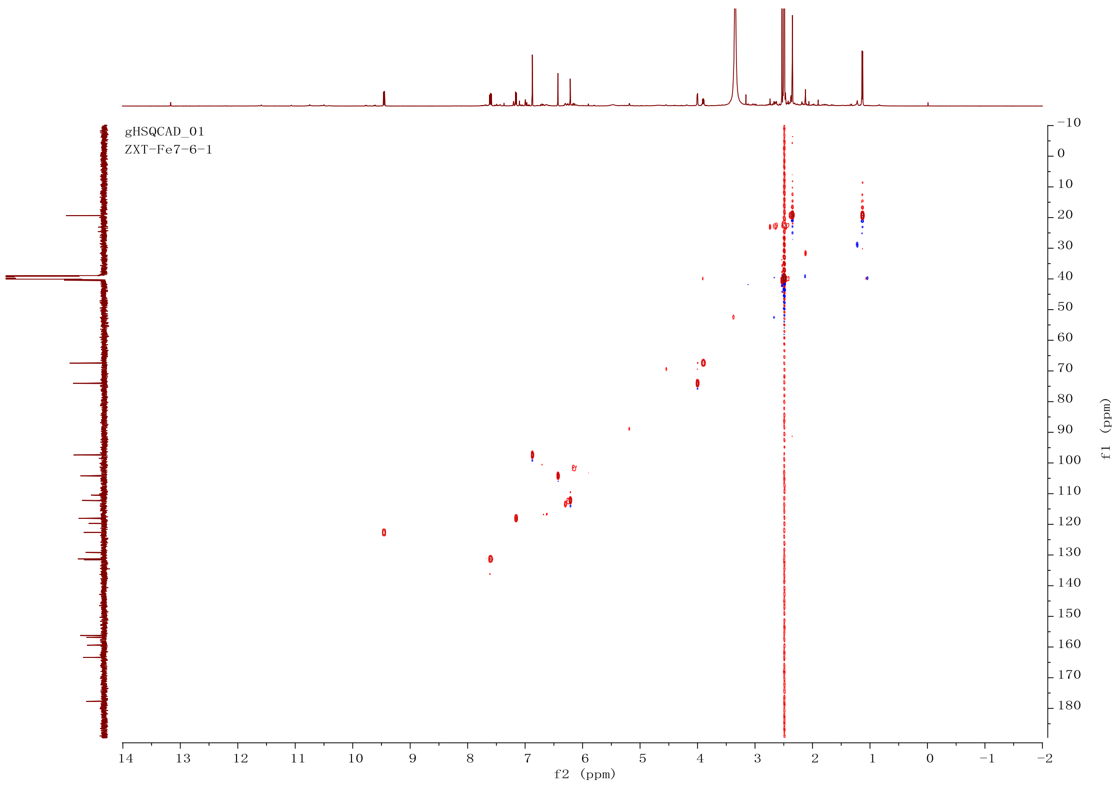

Figure S19 HSQC spectrum of naphpyrone K (**3**) in DMSO-d_6

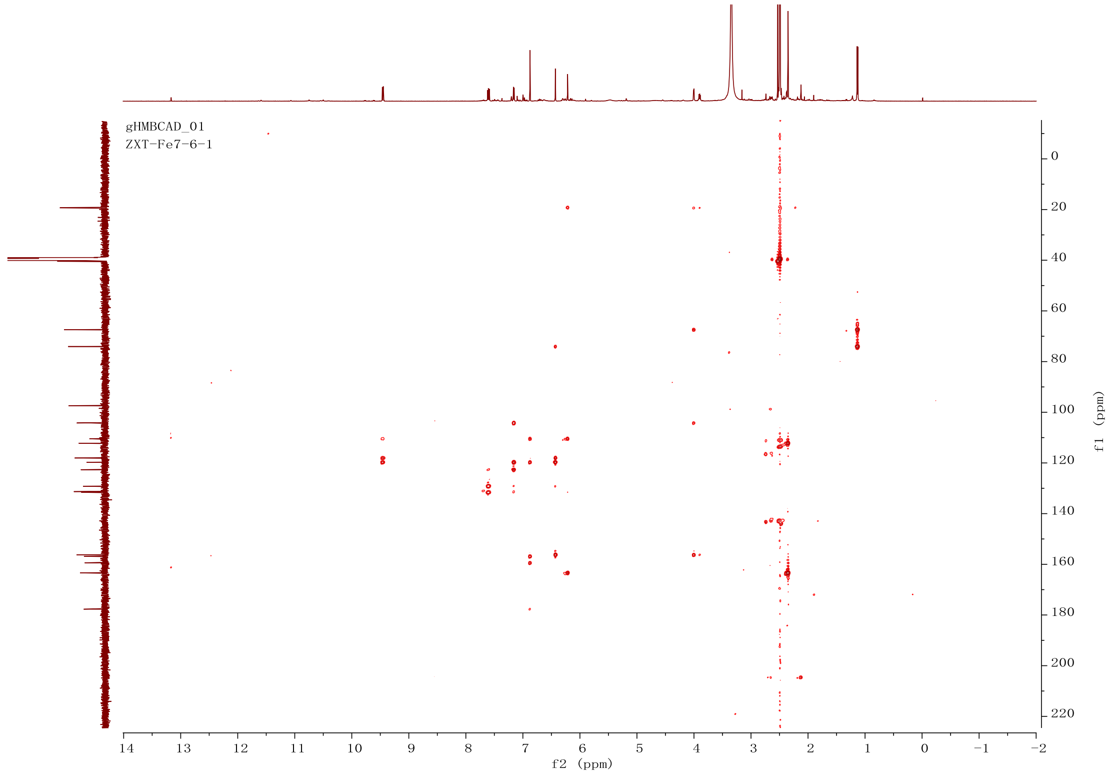

Figure S20 HMBC spectrum of naphpyrone K (**3**) in DMSO-d_6

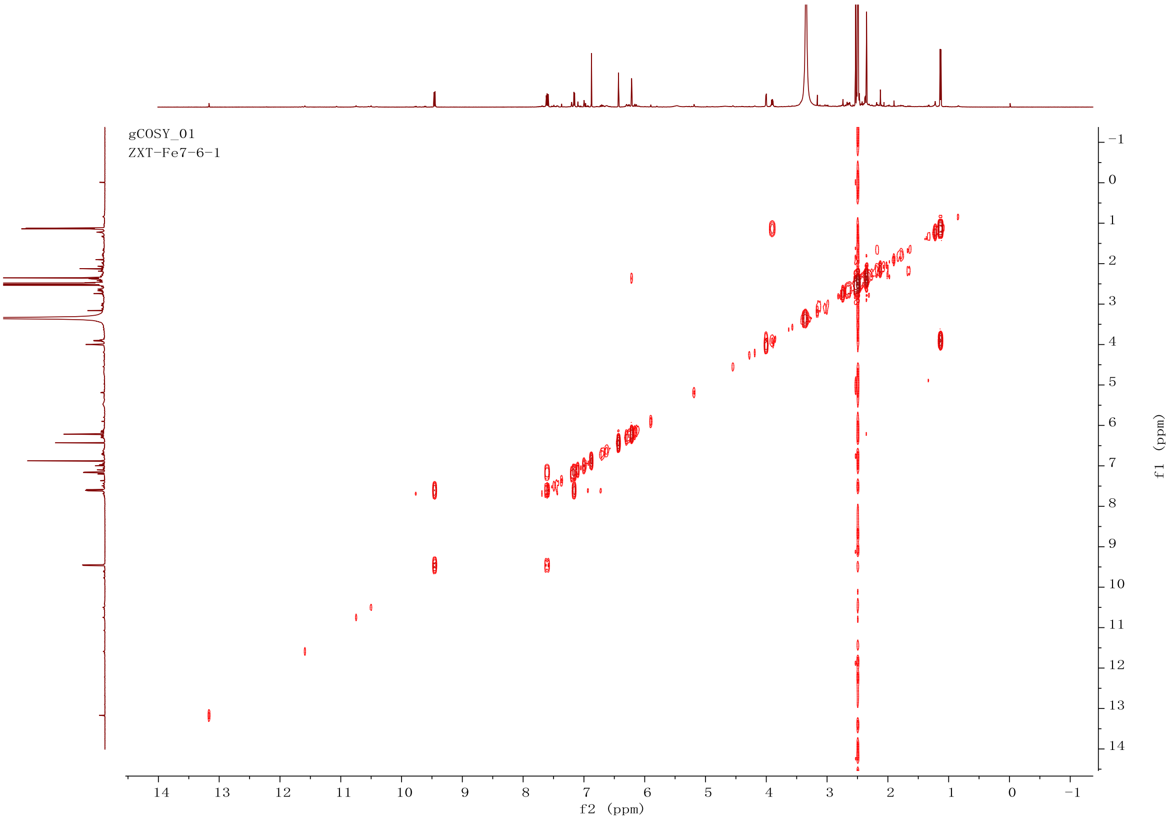

Figure S21 ^{1}H-^{1}H COSY spectrum of naphpyrone K (**3**) in DMSO-d_6

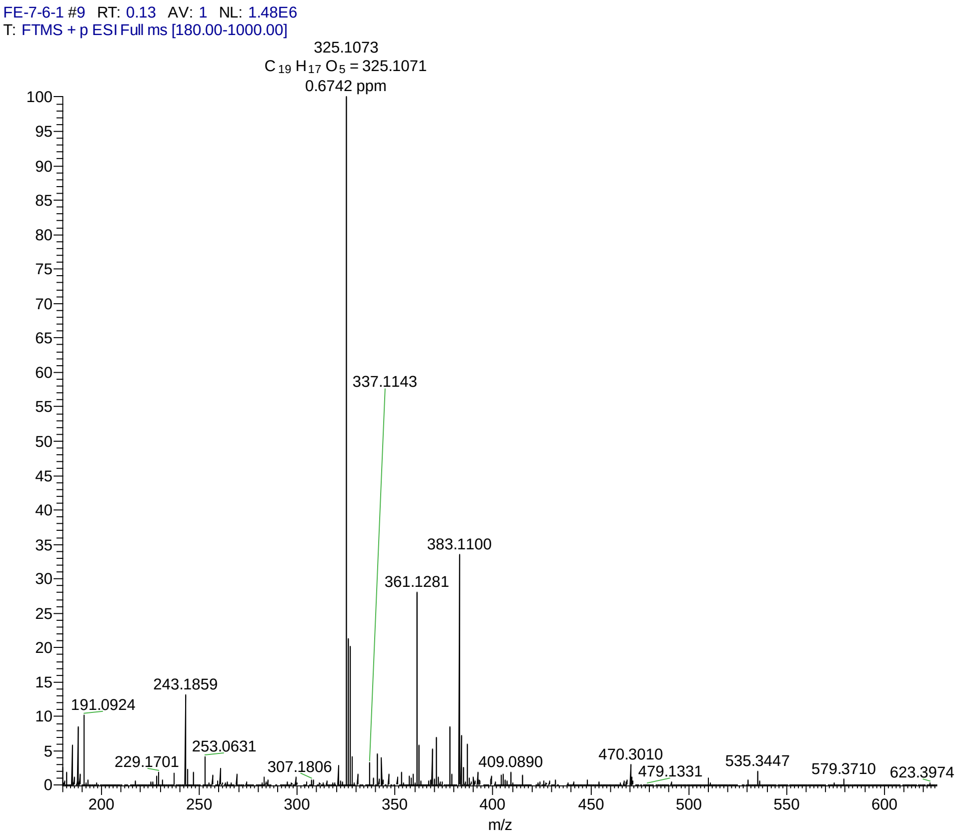

Figure S22 HRESIMS spectrum of naphpyrone K (**3**)
